# Overcoming Anatomical Challenges: Laparoscopic Cholecystectomy in Situs Inversus Totalis

**DOI:** 10.7759/cureus.46371

**Published:** 2023-10-02

**Authors:** Daniel A Meza-Martinez, Julio A Palomino-Payan, Samantha J Andrade-Ordoñez, Brando J Fematt-Rodriguez, Marco Antonio Muñuzuri-Camacho, Alberto Gonzalez-Quezada

**Affiliations:** 1 General Surgery, Instituto Mexicano del Seguro Social, Hospital General de Zona No. 33, Monterrey, MEX; 2 Neurosurgery, Instituto Nacional de Neurología y Neurocirugía Manuel Velasco Suárez, Ciudad de Mexico, MEX

**Keywords:** laparoscopic cholecystectomy, difficult laparoscopic cholecystectomy, diagnostic laparoscopy, complete situs inversus, chronic cholelithiasis, situs inversus totalis

## Abstract

Situs inversus totalis (SIT) is a rare genetic condition characterized by the sagittal inversion of thoracoabdominal organs. Surgeons may face substantial challenges when dealing with surgical pathologies in SIT patients, particularly those involving the gallbladder and bile ducts, such as cholelithiasis and acute cholecystitis. In this report, we present the case of a 46-year-old male with a previously known diagnosis of SIT, who presented with recurrent episodes of atypical abdominal pain. Cholelithiasis was diagnosed through ultrasound and as a result, elective surgery was scheduled. In addition, we detail the adjustments implemented by our surgical team in the laparoscopic cholecystectomy procedure, which contributed to a successful surgical outcome. Nevertheless, like any patient, those with SIT are not exempt from postoperative complications, as detailed in this case. Hence, we emphasize the importance of comprehensive preoperative diagnostics to reduce the risk of perioperative complications in this group of patients.

## Introduction

Situs inversus totalis (SIT) is an exceedingly rare genetic condition characterized by the inversion of thoracoabdominal organs along the sagittal plane [1]. This rearrangement results in a 'mirror image' appearance of the thoracic and abdominal organs [2]. The alteration in organ positioning relative to the sagittal plane arises from anomalies during gastrulation in the embryonic phase. Although the exact underlying causes remain incompletely understood, this condition has been associated with genetic mutations on chromosomes 7, 8, and 14 [3]. It has also been documented to exhibit an autosomal recessive inheritance pattern [3,4].

SIT affects less than 0.01% of the population, occurring in approximately 1 out of every 10,000 to 20,000 live births. While the presence of SIT itself does not predispose individuals to any specific diseases, it can complicate the diagnostic approach to common pathologies, presenting unique challenges for healthcare providers [1,2,5]. In patients with this anomaly, the clinical presentation of cholelithiasis and cholecystitis occurs at a rate comparable to that observed in the general population. The altered anatomy can lead to signs and symptoms that may be misleading or deceptive. When surgical intervention becomes necessary, it is crucial to recognize that their condition demands a more complex surgical approach and a high level of surgical expertise to effectively address these rare scenarios [2].

Given that this rare condition further complicates an already challenging intervention, and considering that various surgical approaches have been documented for this patient population, we aim to share our experience in managing a patient with SIT and cholelithiasis. Within this context, we highlight the modifications and technical adaptations employed in our case, ultimately leading to a successful laparoscopic cholecystectomy, despite technical challenges.

## Case presentation

A 46-year-old male with a known diagnosis of SIT presented to the outpatient department with abdominal pain. His medical history includes ongoing cardiology follow-up for dyslipidemia, which is managed with atorvastatin. In his most recent cardiology consultation, he underwent a stress test that yielded normal results. Apart from this, he has no other significant medical history.

**Figure 1:**
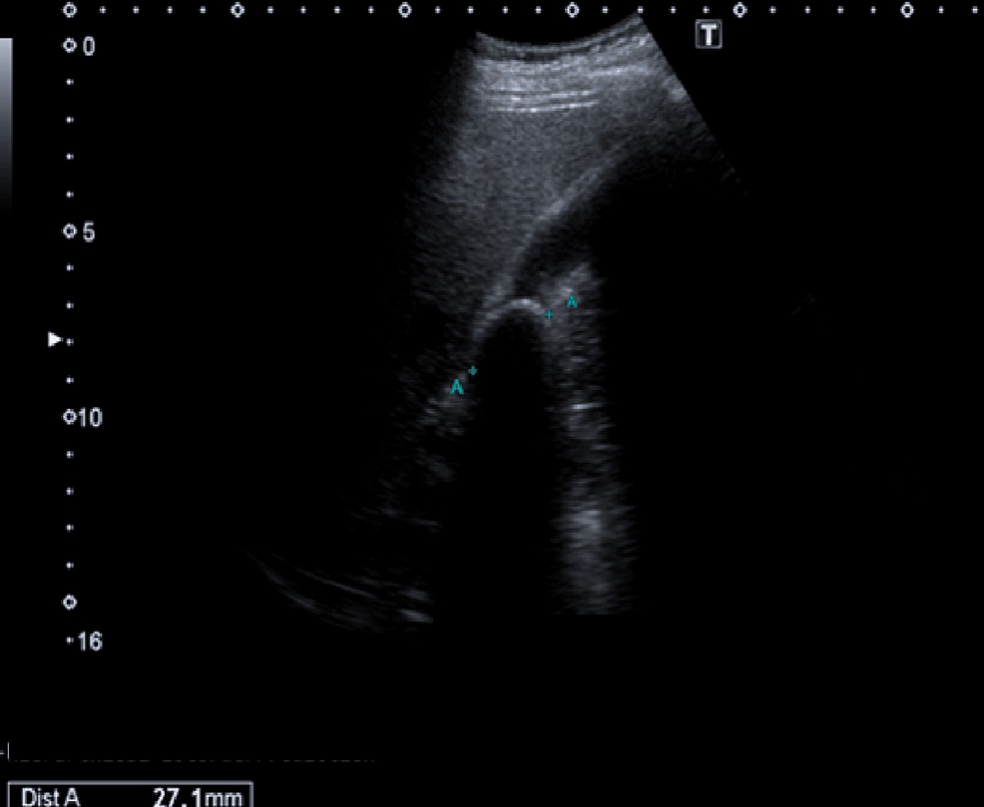
Abdominal ultrasound that confirms the diagnosis of cholelithiasis, revealing a 27-mm gallstone within the gallbladder. No radiological signs of acute cholecystitis were evident during the study. The condition of situs inversus was reaffirmed by the radiologist as an added comment on his ultrasonographic report, as he added the following note: "Patient with a history of SIT, so the liver and gallbladder are visualized in the upper left hemiabdomen, and the spleen in the upper right hemiabdomen."

One year prior to his surgical evaluation, he began experiencing intermittent epigastric abdominal pain with radiation to the left thoracic region. He reported a deterioration in these symptoms one month before seeking assessment by our surgical team. Following a thorough medical assessment, he denied experiencing any other symptoms. During the physical examination, the only notable finding was tenderness in the upper left side of the abdomen. Murphy's sign was negative, and no other abnormalities were detected during the examination. An abdominal ultrasound was conducted, revealing the presence of a 27-mm gallstone, with no signs of acute cholecystitis (Figure 1).

After confirming the presence of cholelithiasis, the patient underwent a preoperative assessment by the internal medicine department, which determined a low cardiovascular risk and identified no contraindications for surgery. During the preanesthetic consultation, the patient reported no respiratory symptoms, and preoperative laboratory studies, such as complete blood count, comprehensive metabolic panel, liver function tests, and serum lipase, all resulted within normal ranges. Based on this information, a laparoscopic cholecystectomy was scheduled due to recurrent pain episodes and the size of the gallstone.

**Figure 2:**
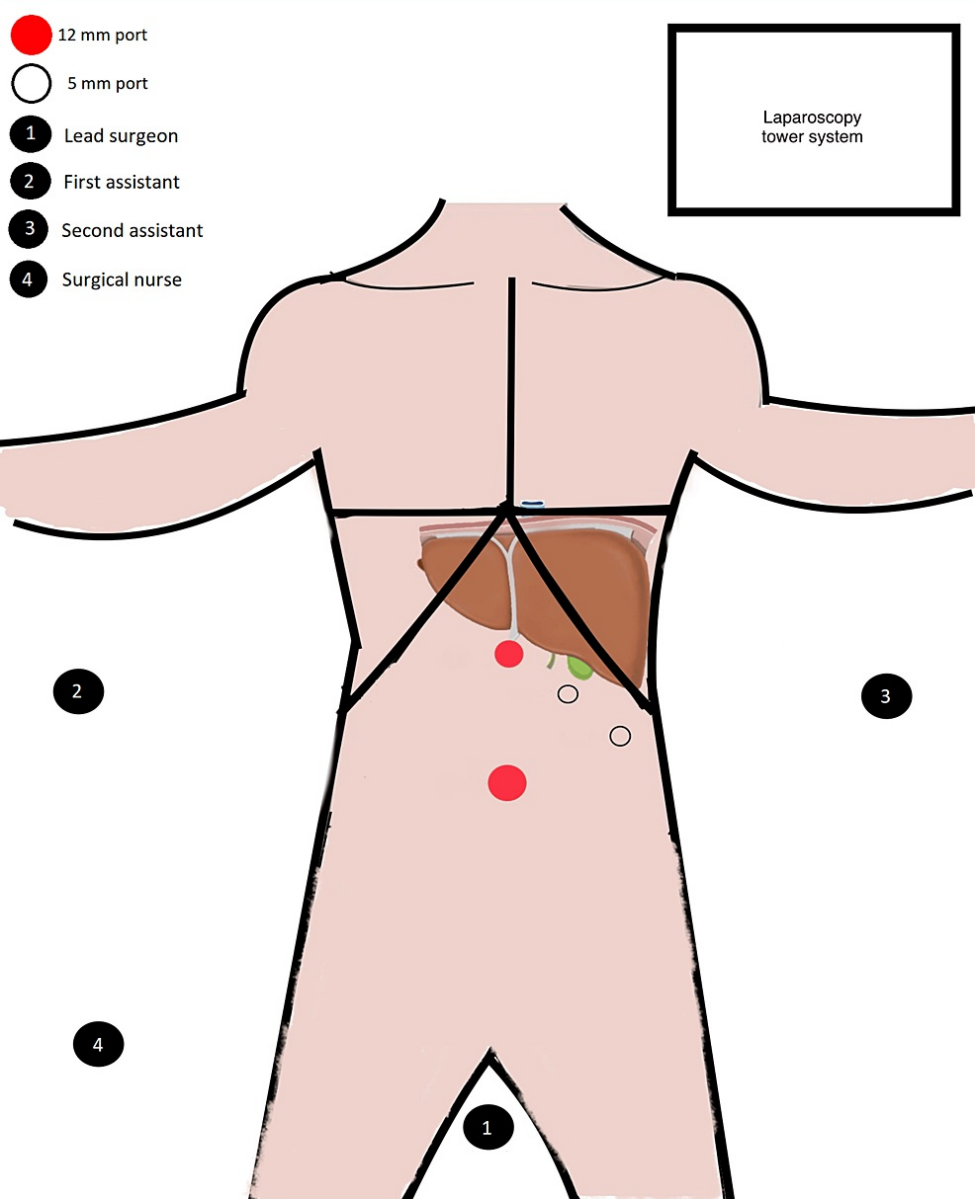
Operating room setup and positioning. Diagram that illustrates the operating room layout, showing a mirror-image of laparoscopic cholecystectomy performed using the American technique. However, in this instance, the surgeon is positioned between the patient's abducted legs, following the French technique.

On the day of surgery, the anesthesiology department reported no anesthetic-related complications or difficulties with intubation prior to the procedure's commencement. Due to the presence of SIT, we modified the standard surgical procedure typically performed in our unit. This adjustment required reconfiguring both the operating room layout and the positioning of the surgical team, creating a mirrored setup compared to the usual configuration. The primary modification centered around the lead surgeon's positioning, who placed himself between the patient's abducted legs, following the approach described in the French technique (Figure 2).

**Figure 3:**
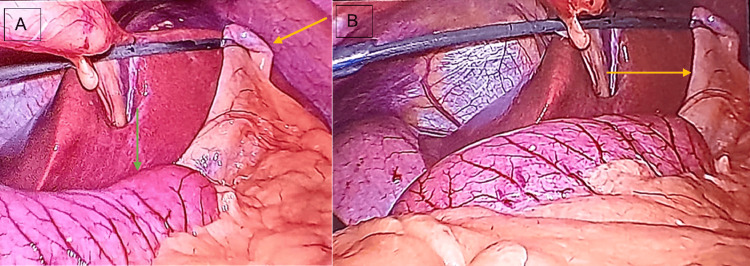
Diagnostic laparoscopy A: Laparoscopic image demonstrating the presence of abdominal situs inversus, showing the liver and gallbladder in the left hypochondrium (yellow arrow) and the stomach positioned towards the midline and right hypochondrium (green arrow). B: After retracting the gallbladder fundus, omental adhesions obscuring the majority of the gallbladder can be seen (yellow arrow), categorizing it as grade 4 on the Parkland Grading Scale for cholecystitis.

The procedure began with a transumbilical incision, followed by the insertion of a 12-mm umbilical port, which was used to establish pneumoperitoneum. As the abdominal cavity was insufflated, the laparoscope was introduced. Diagnostic laparoscopy showed the liver and the gallbladder located in the left hypocondrium, with a gallbladder obscured by omental adhesions (Figure 3).

Following this, the patient was repositioned laterally to the right, and, with direct visualization, a 5mm trocar was inserted at the left anterior axillary line, positioned 3 cm below the costal margin. Subsequently, another 5-mm port was situated at the left midclavicular line, equidistant from the preceding ports. Finally, a fourth 12-mm trocar was introduced, positioned midway between the xiphoid process and the umbilicus.

**Figure 4:**
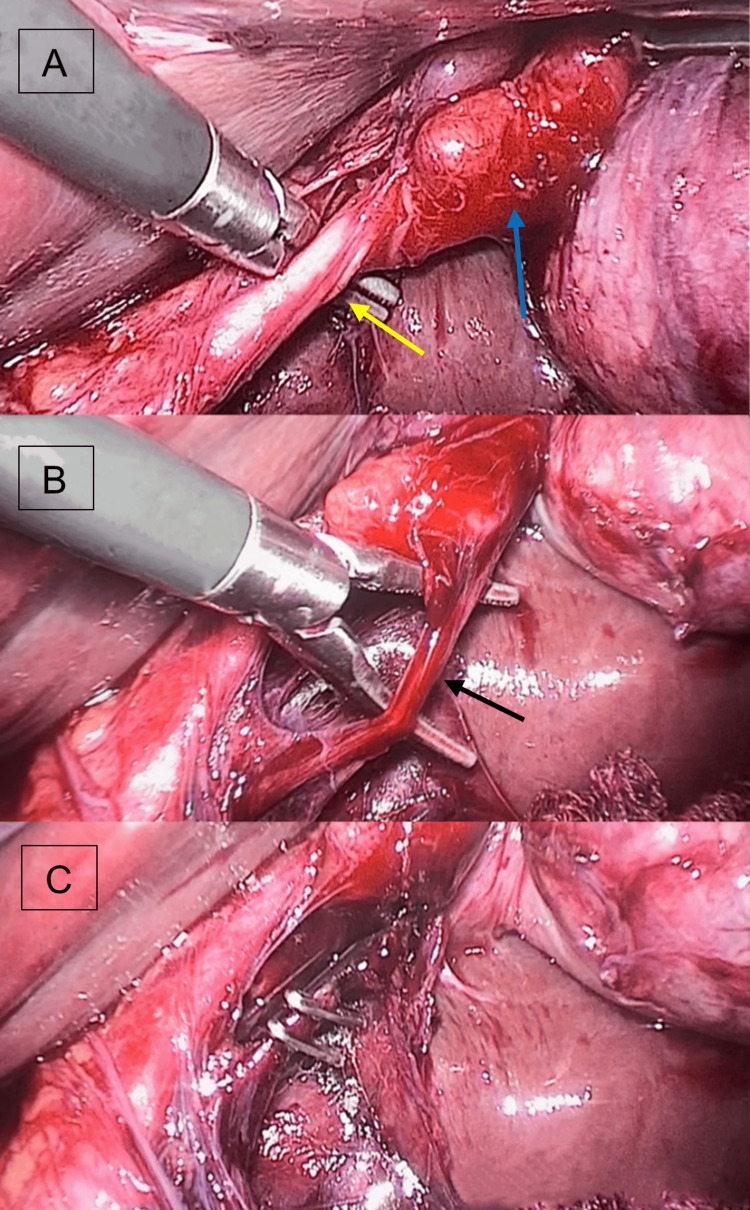
Laparoscopic images illustrating the handling of structures within the hepatocystic triangle Dissection of the hepatocystic triangle is depicted as follows: A) Dissection of the cystic duct (indicated by the yellow arrow). Additionally, a cystic lymph node is visible (blue arrow); B) Dissection of the cystic artery (black arrow); C) Posterior cystic artery ligation achieved with laparoscopic clips.

Once all ports were appropriately positioned, cephalad traction was applied through the anterior axillary port, with the second assistant grasping the gallbladder's fundus to enhance visibility for the lead surgeon. Subsequently, omental adhesions were released, and the surgery proceeded with dissection of the hepatocystic triangle. The lead surgeon applied traction to Hartmann's pouch with his right hand through the midclavicular port. Employing the epigastric 12-mm port, structural dissection was performed. Once the anatomical structures of the hepatocystic triangle were identified, laparoscopic clips were employed to individually ligate each element (Figure 4). The artery and cystic duct were transected, and the gallbladder dissection was completed, subsequently extracting it from the abdominal cavity through the epigastric port. Hemostasis was confirmed, along with the integrity of the remaining organs and the bile ducts, concluding the procedure.

**Figure 5:**
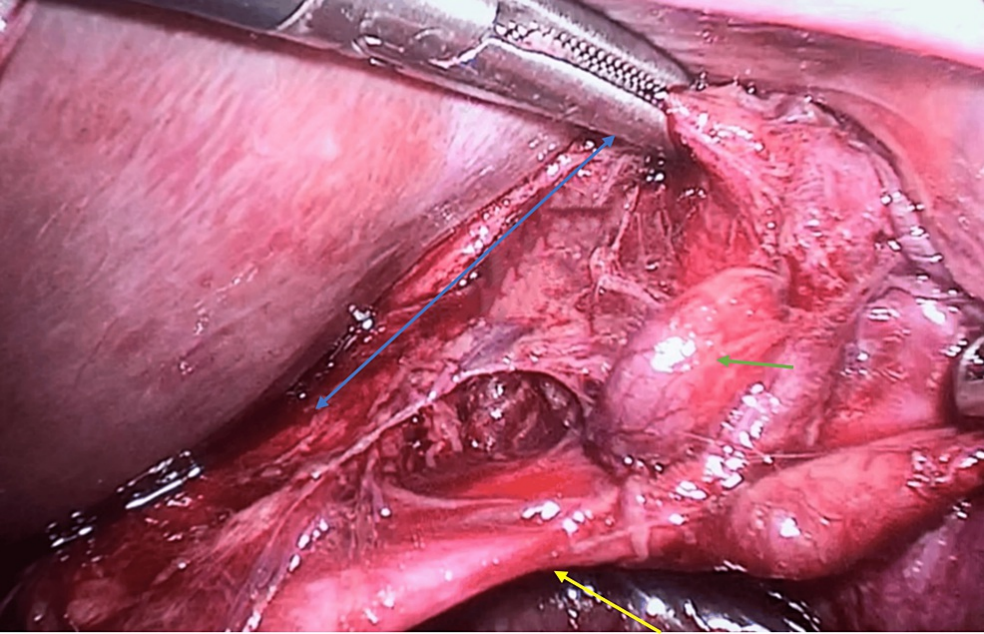
Laparoscopic image showing the anterior critical view of safety. Due to the anatomical alteration and procedure complexity, the lead surgeon chose to ligate the cystic artery first.* The image displays the cystic duct (highlighted by the yellow arrow) and partial visibility of the cystic plate (indicated by the green arrow), as well as the cystic lymph node (blue arrow). No anatomic variations of the cystic artery or biliary tract were detected. *: cystic artery not shown in this image; refer to Figure 4 for cystic artery ligation.

Ultimately, laparoscopic findings revealed a thin-walled gallbladder, which measured 8 x 4 x 4 cm, and contained a 2-cm gallstone. A 3-mm posterior cystic artery with a 4-mm cystic duct were identified. No substantial abnormalities within the vasculature or biliary tract were observed (Figure 5).

**Figure 6:**
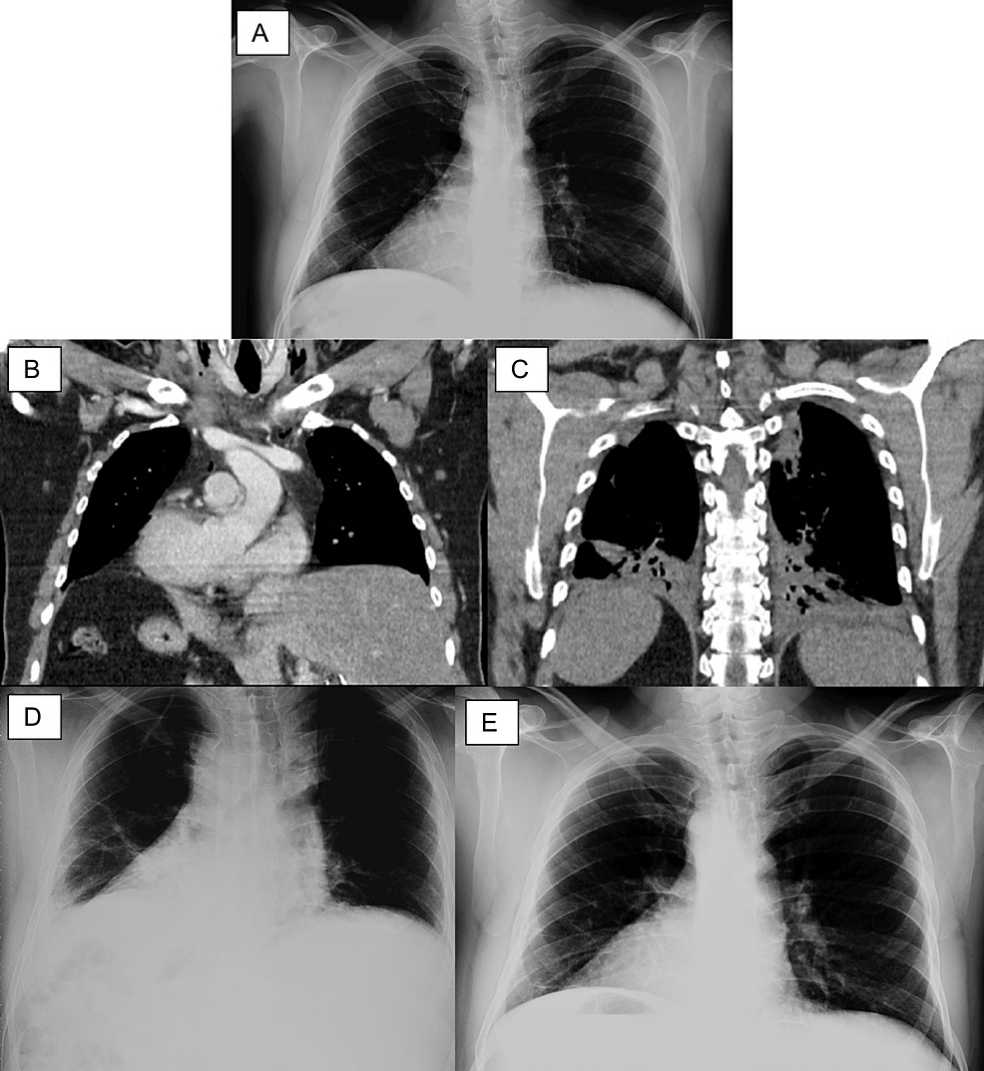
Pre and postoperative chest x-rays and simple and contrast-enhanced thoracoabdominal CT with postoperative pulmonary alterations. A) Preoperative chest x-ray that shows dextrocardia; B) Simple and contrast-enhanced thoracoabdominal CT with postoperative pulmonary alterations. Coronal section with a middle mediastinal window showing dextrocardia, with the liver situated in the left hypochondrium and the stomach in the right hypochondrium, this confirms the diagnosis, not only of situs inversus, but of SIT, as a complete transposition of thoracoabdominal organs is shown; C) Same CT scan, in a posterior coronal section showing the presence of incipient bilateral pleural effusion, bilateral posterobasal consolidations and basal atelectasis; D) Postoperative chest x-ray 48 hours after conservative management with respiratory exercises and oxygen therapy, showing a significant radiographic improvement with persistent right pleural effusion; E) Follow-up chest x-ray demonstrating resolution of atelectasis and pleural effusion. CT: computed tomography

Once the procedure concluded, he was transferred to the post-anesthesia recovery room. Approximately two hours after the surgery, the patient developed respiratory difficulty characterized by tachypnea, with a rate of up to 30 breaths per minute, and desaturation as indicated by pulse oximetry, with oxygen saturation dropping to 88%. Consequently, he was admitted to the hospital for monitoring and management. A simple and contrast-enhanced thoracoabdominal tomography was requested, which revealed incipient bilateral pleural effusion and the presence of bilateral posterobasal consolidations associated with basal atelectasis. He received conservative management, which included oxygen therapy and breathing exercises for two days. During the mid-post-surgical period, he experienced an adequate clinical course, with his symptoms resolving completely. Consequently, he was discharged home three days after his surgery. A month later, at his follow-up appointment, he was permanently discharged, as he remained asymptomatic and his follow-up chest x-ray showed a complete resolution of pleural effusion and atelectasis when compared to previous imaging studies (Figures 6A-6E).

## Discussion

The presence of SIT entails a complete transposition of thoracic and abdominal organs. Discrepancies related to terminology exist; however, it is generally accepted to consider situs solitus as representative of normal anatomy, SIT for complete reversal of thoracoabdominal organs, and 'situs ambiguous' to encompass a wide spectrum of anatomic variations, where there are multiple alterations in the left-right arrangement of organs but cannot be classified as SIT [5].

In terms of its pathogenesis, paired like homeodomain transcription factor 2 (Pitx2) plays a vital role during embryogenesis, establishing the left-right axis in the lateral mesoderm and contributing to the asymmetric development of organs like the heart, small intestine, and stomach. When Pitx2 is deleted, it disrupts the normal organ morphogenesis, leading to asymmetrical development on the left side of specific visceral organs, including the spleen and liver [6]. However, it is important to note that pathologies such as intestinal malrotation, a congenital anomaly of intestinal positioning, differs from SIT. While the liver, bile ducts, gallbladder, esophagus, stomach, duodenum, and pancreas develop from the foregut and do not rotate during embryonic development, the small intestine, cecum, ascending colon, and transverse colon develop from the midgut and undergo a 270° counterclockwise rotation around the superior mesenteric artery. Intestinal malrotation occurs due to an error in this process and is therefore independent of SIT [5].

Nevertheless, the presence of situs anomalies can be accompanied by various comorbidities [6]. In individuals with situs solitus, the prevalence of congenital heart disease stands at approximately 0.6%. In contrast, for those with SIT, this rate varies from 3% to 9%, and it climbs to nearly 80% in cases of situs ambiguous. Additionally, vascular anomalies, such as an interrupted inferior vena cava and a preduodenal portal vein, have been documented in up to 20% to 42% of patients with situs abnormalities, respectively. Furthermore, the occurrence of an aberrant hepatic artery is more common among patients with abnormal situs [5,6]. Biliary tract malformations are relatively common, occurring in 5.2% of cases, with biliary atresia being the most frequent [7]. In our case, the patient fulfilled the diagnostic criteria for SIT, exhibiting a sagittal inversion of both abdominal and thoracic organs, without any additional anatomical abnormalities in vascularization or the biliary tract.

Despite the aforementioned, there is no evidence that patients with SIT are predisposed to cholelithiasis [8]. Physical examination findings can be deceptive in patients with SIT. In cases of acute cholecystitis, these patients might complain of upper left quadrant abdominal pain, occasionally accompanied by radiation to the ipsilateral scapula. This pain is a result of peritoneal irritation. Conversely, when there is no peritoneal irritation, such as in biliary colic, the pain may be localized to the epigastrium [9]. A cardiac apex beat in the fifth right intercostal space, as well as the absence of dullness on percussion in the right hypochondrium, should raise diagnostic suspicion [2]. Because of the alteration in anatomical position, the clinical presentation may resemble that of acute pancreatitis [10]. Ultrasound, tomography, chest X-ray, and magnetic resonance imaging will confirm the presence of visceral transposition [2].

Much like what has been documented in the literature, the patient in question reported pain radiating to the left hemithorax, an atypical presentation in gallbladder pathology that should prompt the examiner's attention. In such instances, there is a potential concern for an undiagnosed heart condition. However, in our case, the preoperative evaluation revealed no indications of cardiopulmonary disease, aligning with the findings from the previous cardiological assessment. Similarly, the likelihood of acute biliary pancreatitis was excluded through comprehensive laboratory and imaging studies.

To provide appropriate care in emergency services, it is necessary to conduct a comprehensive and detailed diagnostic approach and maintain a high index of suspicion. It's worth noting that performing an urgent surgical procedure carries the risk of encountering unexpected anatomical barriers. Detecting anatomical inversion during surgery can lead to serious complications [11]. Before the advent of minimally invasive surgery, up to 40 cases of conventional cholecystectomy in patients with SIT were reported, yielding satisfactory results and a low rate of complications [12]. The presence of SIT is not a contraindication for laparoscopic cholecystectomy. The procedure is considered safe and, in fact, is now the standard of treatment. Nevertheless, it poses technical challenges due to the mirror-image anatomy, necessitating precise dissection of the biliary tree to avoid iatrogenic injuries [13].

Surgery in patients with SIT presents a significant challenge due to the structural anatomical inversion. During the procedure, the change in the orientation of the gallbladder and its structures complicates dissection. One of the most crucial steps is the proper placement of trocars to attain an adequate critical view of safety and minimize the risk of bile duct injuries [14]. To address this challenge, some surgeons choose to position a second assistant on the patient's left side, who can hold and retract the gallbladder infundibulum while they position themselves on the patient's right side and perform dissection [15]. There have also been documented reports in the literature regarding the utilization of the French technique. This approach may necessitate the surgeon, especially if they are right-handed, to conduct anatomical dissection using their non-dominant hand [7]. In other instances, surgeons prefer to retract the gallbladder infundibulum through the epigastric port using their left hand while employing their right hand for dissection through the left midclavicular port. This procedure is typically performed with the patient in a supine position [16]. There is no standardized technique for these cases. The surgeon should adapt the procedure based on their judgment to enable meticulous dissection and attain a critical view of safety [13].

The technique employed by our surgical team consisted of a combination of previously described approaches in the literature and from prior case reports. On this occasion, the operating room setup and team members followed the mirror-image orientation of the standard American laparoscopic cholecystectomy technique, which is routinely used in our hospital. However, the primary difference was that the surgeon positioned themselves in accordance with the French technique, placing themselves between the abducted legs of the patient. This positioning allowed for the surgeon to perform using both hands. As a result, the dissection and ligation of the cystic artery and duct were performed using the left hand, while the right hand provided anterior, posterior, and lateral traction of the infundibulum, alternating between these positions to maintain an optimal dissection plane, which provided a clearer view of biliary anatomy. The surgeon’s first assistant, who was situated to the right of the patient, manipulated the laparoscopy lens. It’s worth highlighting that the surgeon had a second assistant, who was positioned to the left of the patient and was responsible for cephalad traction of the gallbladder fundus, aiding the surgeon’s vision.

When it is not feasible to achieve an optimal critical view of safety due to the patient's anatomical alterations, the use of intraoperative cholangiography has been recommended as a means to enhance the visualization of biliary structures [7,16]. Regarding the previous statement, high success rates have been demonstrated in bile duct exploration for patients with SIT and choledocholithiasis, a complication of cholelithiasis that can occur in any patient, regardless of SIT. No major complications have been reported associated with the use of intraoperative cholangiography or endoscopic retrograde cholangiopancreatography (ERCP) for the resolution of choledocholithiasis or to clarify bile duct anatomy in this group of patients [2]. One of the limitations in this case was the inability to conduct an intraoperative cholangiography due to the unavailability of contrast in the hospital. Intraoperative cholangiography is a valuable study for precisely assessing the anatomy of the bile ducts and is recommended when achieving a critical view of safety is not attainable, like in this case.

It's essential to highlight that these patients have a higher risk of anesthetic complications, primarily due to the elevated incidence of congenital heart diseases. Therefore, it is imperative to exercise caution and implement specific precautions during anesthetic management. Reports show a greater predisposition for selective intubation of the left bronchus; if the patient has abnormalities of the great vessels, preference should be given to cannulation of the left jugular vein to avoid injury to other vascular structures, thus ensuring direct access to the right atrium [17]. Likewise, it is advisable to evaluate the patient for other cardiovascular and pulmonary conditions during the preoperative phase whenever feasible [18]. A proper diagnosis and a thorough study prior to intervention can help prevent surgical, pulmonary, and cardiac complications in these patients. Likewise, the complete and detailed evaluation allows the surgeon and his team to prepare mentally, ergonomically and physically, given the inversion in the placement of medical staff and instruments, as well as mitigates the cognitive dissonance caused by the inverted anatomy of the abdominal organs [19].

Our patient did not experience complications during the surgical event. However, the appearance of respiratory symptoms in the immediate postoperative period is in line with what is described in the literature. Factors such as fluid overload administered by the anesthesiology service, exacerbation of underlying, undiagnosed cardiac conditions, or the presence of postoperative complications such as pulmonary embolism could account for what occurred in this case. Given the clinical presentation, it was essential to rule out pulmonary embolism. One challenge in this situation was the unavailability of a CT pulmonary angiogram (CTPA) at our institution; therefore, we could only perform a contrast-enhanced thoracoabdominal CT. We consider this as a limitation in our case, as CTPA is a more diagnostically certain study and the preferred choice for such diagnostic suspicion. Fortunately, the contrast-enhanced thoracoabdominal CT, coupled with the patient's rapid clinical improvement, allowed us to rule out pulmonary embolism. After ruling out the more severe pathology, it is possible that the patient's clinical deterioration was a result of fluid overload or atelectasis. Both of these are common complications in these types of procedures and could similarly account for the respiratory symptoms observed in this case.

## Conclusions

Laparoscopic cholecystectomy is considered a safe and effective treatment for patients with SIT. However, both literature reports and our own case experience have emphasized that the unique anatomical arrangement in SIT necessitates modifications in the surgical technique, thus requiring a surgeon with appropriate training who possesses an in-depth understanding of biliary anatomy and its variations. In our case, prior knowledge of the patient's diagnosis of SIT allowed us to conduct a thorough preoperative evaluation, ultimately leading to a challenging but successful surgical outcome. Nonetheless, the patient did experience postoperative pulmonary complications. This underscores the significance of contemplating cardiopulmonary screening for SIT patients as a potential measure to mitigate perioperative morbidity and mortality, as well as postoperative complications.
